# Supramolecular Electrochemical Sensor for Dopamine Detection Based on Self-Assembled Mixed Surfactants on Gold Nanoparticles Deposited Graphene Oxide

**DOI:** 10.3390/molecules25112528

**Published:** 2020-05-29

**Authors:** Pikaned Uppachai, Supalax Srijaranai, Suta Poosittisak, Illyas Md Isa, Siriboon Mukdasai

**Affiliations:** 1 Department of Applied Physics, Faculty of Engineering, Rajamangala University of Technology Isan, Khon Kaen Campus, Khon Kaen 40000, Thailand; pikaned.up@rmuti.ac.th; 2 Materials Chemistry Research Center, Department of Chemistry and Center of Excellence for Innovation in Chemistry, Faculty of Science, Khon Kaen University, Khon Kaen 40002, Thailand; supalax@kku.ac.th (S.S.); anthony@kku.ac.th (S.P.); 3 Department of Chemistry, Faculty of Science and Mathemathics, Universiti Pendidikan Sultan Idris, 35900 Tanjong Malim, Perak, Malaysia; illyas@fsmt.upsi.edu.my

**Keywords:** dopamine, gold nanoparticles, graphene oxide, glassy carbon electrode, supramolecular electrochemical sensor

## Abstract

A new supramolecular electrochemical sensor for highly sensitive detection of dopamine (DA) was fabricated based on supramolecular assemblies of mixed two surfactants, tetra-butylammonium bromide (TBABr) and sodium dodecyl sulphate (SDS), on the electrodeposition of gold nanoparticles on graphene oxide modified on glassy carbon electrode (AuNPs/GO/GCE). Self-assembled mixed surfactants (TBABr/SDS) were added into the solution to increase the sensitivity for the detection of DA. All electrodes were characterized by scanning electron microscopy (SEM), cyclic voltammetry (CV), and electrochemical impedance spectroscopy (EIS). The supramolecular electrochemical sensor (TBABr/SDS⋅⋅⋅AuNPs/GO/GCE) showed excellent electrocatalytic activity toward the oxidation of DA. Under the optimum conditions, the concentration of DA was obtained in the range from 0.02 µM to 1.00 µM, with a detection limit of 0.01 µM (3s/b). The results displayed that TBABr/SDS⋅⋅⋅AuNPs/GO/GCE exhibited excellent performance, good sensitivity, and reproducibility. In addition, the proposed supramolecular electrochemical sensor was successfully applied to determine DA in human serum samples with satisfactory recoveries (97.26% to 104.21%).

## 1. Introduction

Dopamine (3,4-dihydroxyphenyl ethylamine, DA) is a neurotransmitter that plays an important role in communication in the central nervous system. Low levels of DA may cause neurological disorders including Alzheimer’s and Parkinson’s [1,2]. Thus, the accurate and rapid determination of DA concentration in body fluid is of great significance. Currently, several techniques have been reported for the detection of DA such as spectrophotometry [3,4,5], high performance liquid chromatography [6,7], and electrochemistry [8,9,10]. Among these methods, the electrochemical technique has gained much interest because of its simplicity, rapidity, high sensitivity, and low operation cost. However, common interfering compounds ascorbic acid (AA) and uric acid (UA) usually coexist with DA in the human body [11,12,13,14], and can interfere with the response of DA. The overlapping of the oxidation potentials of DA, AA, and UA at bare solid electrodes also causes trouble for their simultaneous determination. Therefore, to improve the sensitivity of DA, the nanomaterials-based electrodes were required.

Many types of materials have been employed to modify electrodes such as metal nanoparticles [15,16,17,18], conducting polymer [19,20,21,22], and carbon-based materials [23,24,25,26] to fabricate the highly selective and sensitive of DA. Graphene has sp^2^-hybridization carbon materials, including graphene oxide (GO) and reduced graphene oxide (rGO). They have gained enormous attention for use as a modifier on the surface of electrode owing to their high electronic conductivity and electrocatalytic activity [27,28], as well as other electrochemical applications including energy storage [29,30]. GO has been applied to fabricate the electrochemical sensors for neurotransmitters [31,32,33,34], but it has a problem on spontaneous aggregation. However, the functionalized GO is needed to improve the solubility of GO [35]. Besides GO, metal nanoparticles are of enormous interest for the modification of electrode [15,36,37,38]. Among all metals, gold nanoparticles (AuNPs) have excellent conductivity and catalytic properties to enhance the electron transfer of electrochemical reactions between target analytes and electrode surfaces. For this reason, the AuNPs deposited on nanomaterials are a good alternative to detect DA [39,40,41,42]. The important properties of the catalyst are strongly affected by the morphology and high dispersion of AuNPs on the surface of GO [43,44].

Supramolecular assembly or supramolecule can be simply produced by self-assembly processes from amphiphiles containing both hydrophobic and hydrophilic parts. Different types of aggregation can be formed such as nanostructures, micelles, or vesicles depending on the composition and shape of amphiphiles in aqueous medium [45]. Surfactants are important amphiphilic compounds that can be generated by the supramolecular assembly and have been used to increase the interface property between the surface of electrode and solution. Supramolecular assemblies have been created by mixing cationic and anionic surfactants into solution. Mixed surfactants exhibit synergism compared with a single surfactant owing to higher surface activity, co-stabilizing, and co-sensitizing properties [45,46]. To the best of our knowledge, the fabrication of a supramolecular assembly between cationic surfactant (tetrabutylammonium bromide, TBABr) and anionic surfactant (sodium dodecyl sulfate, SDS) modified glassy carbon electrode based on gold nanoparticles deposited on graphene oxide (TBABr/SDS⋅⋅⋅AuNPs/GO/GCE) for the detection of DA has not yet been reported.

In the present work, a novel supramolecular electrochemical sensor based on mixed surfactants on gold nanoparticles/graphene oxide modified glassy carbon electrode, TBABr/SDS⋅⋅⋅AuNPs/GO/GCE, was fabricated to detect DA. Morphology and electrochemical properties of the modified electrodes were investigated using scanning electron microscopy (SEM) and electrochemical impedance spectroscopy (EIS). The electrochemical properties of modified electrodes were performed using the differential pulse voltammetry (DPV) technique. The TBABr/SDS⋅⋅⋅AuNPs/GO/GCE electrode showed a high peak and potential for the determination of DA. Moreover, the supramolecular electrochemical sensor showed a high level of sensitivity and excellent performance in stability and reproducibility. The proposed supramolecular electrochemical sensor was applied to human serum for the determination of DA.

## 2. Results and Discussion

### 2.1. Morphological Characterization of the AuNPs/GO/GCE

**Figure 1:**
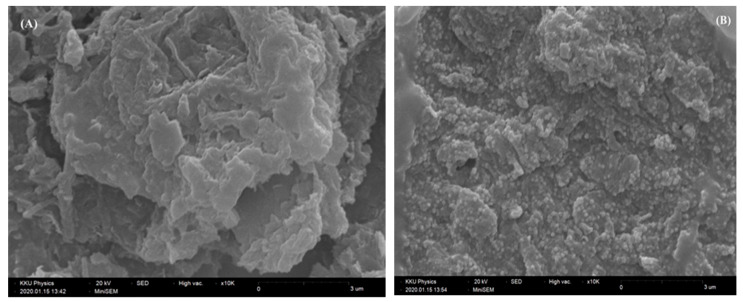
(**A**) Scanning electron microscopy (SEM) image of graphene oxide (GO) and (**B**) AuNPs deposited on GO.

The surface morphology of the AuNPs/GO/GCE was characterized by SEM. Figure 1A shows the irregular shapes of the GO like the aggregation of small pieces of flake [47]. It also indicated that GO has a highly porous nanostructure and electroactive surface to diffuse the analyte into the surface electrode. In Figure 1B, AuNPs were dark dots with a roughly spherical shape and were uniformly distributed on the surface of GO, which confirms the AuNPs deposited on GO.

**Figure 2:**
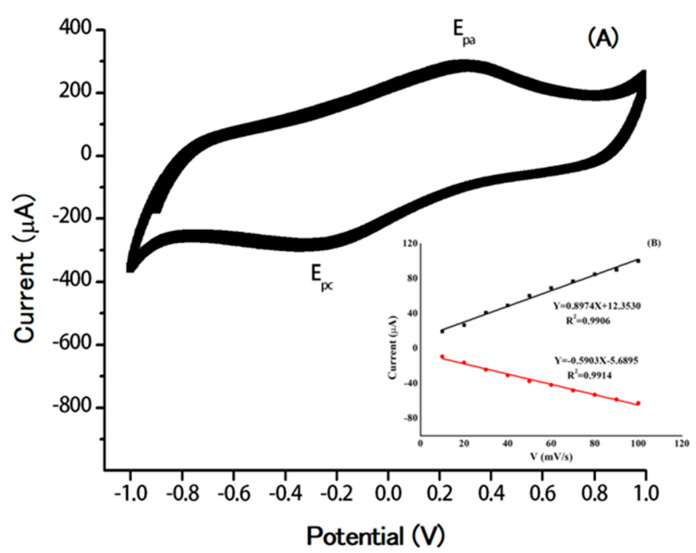
(**A**) Cyclic voltammogram of gold nanoparticles deposited graphene oxide modified on glassy carbon electrode (AuNPs/GO/GCE) in 0.1 M buffer (pH 7.0). Scan rate: 50 mV/s. (**B**) The plot of the anodic and cathodic peak currents vs. the scan rate.

In addition, the accumulation of AuNPs on surface of GO/GCE was confirmed using CV. The modified electrode, AuNPs/GO/GCE, was run in the buffer solution (pH 7.0) for 10 cycles. Figure 2A shows an anodic peak at 0.30 V and a cathodic peak at −0.25 V, which are comparable to the other reports [47,48]. The oxidation peak at 0.30 V refers Au metal to the Au oxide layer, and undergoes reduction at −0.25 (Au oxide to AuNPs) on the surface of electrode. All of the peaks increased, which confirms the deposition of AuNPs on GO/GCE. The scan rate of AuNPs/GO/GCE was also obtained at 10–100 mV/s by the variation of anodic and cathodic peak currents at 0.30 and −0.25 V, respectively (Figure 2B). Both peak potentials increased linearly with the increasing scan rate with a correlation coefficient (R^2^) greater than 0.990, indicating a surface confined process [48].

### 2.2. Electrochemical Characterization of the Modified Electrodes

**Figure 3:**
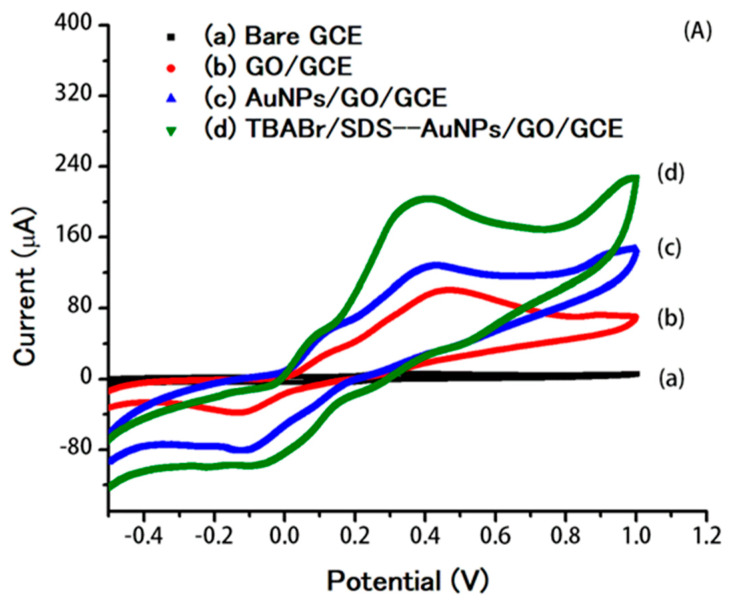

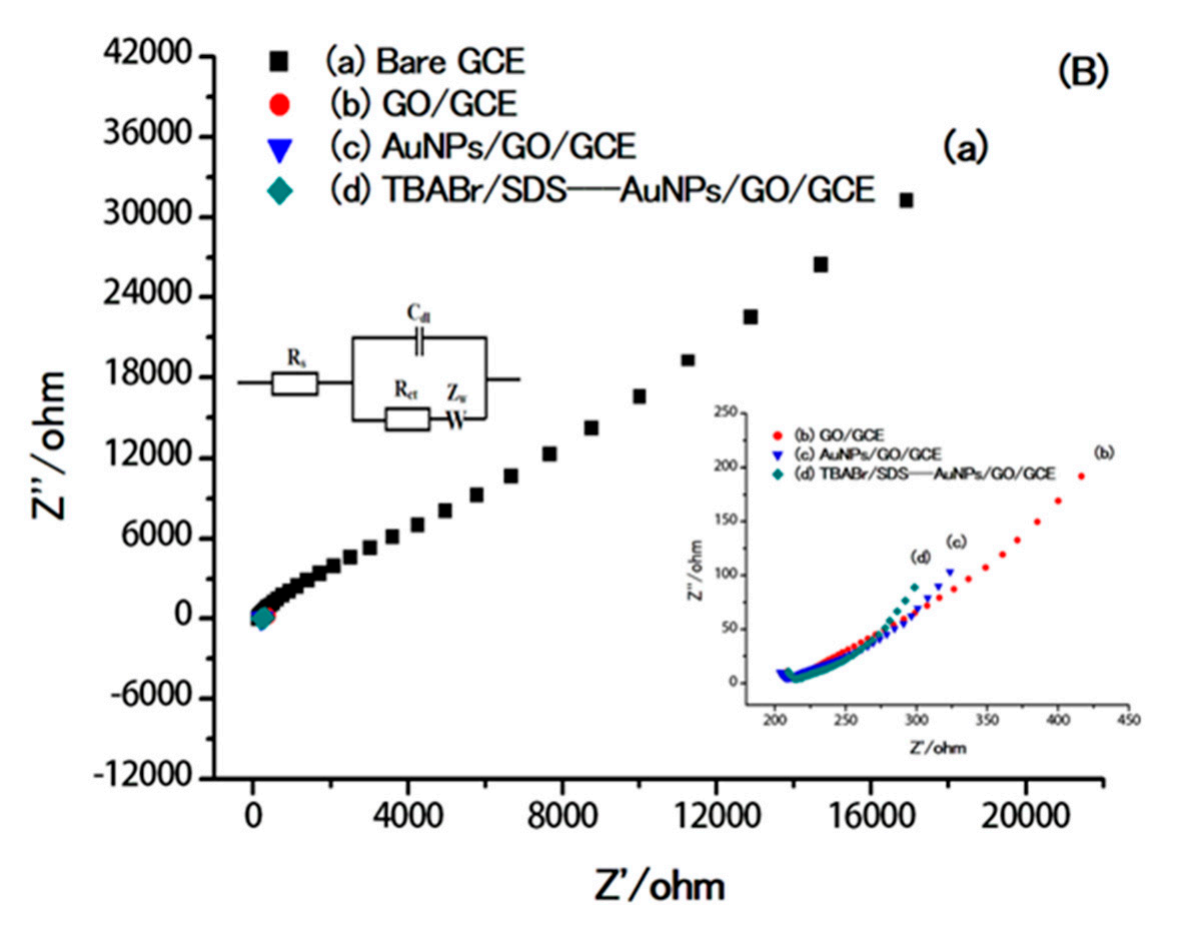
(**A**) Cyclic voltammograms of 0.80 µM dopamine (DA) in 0.1 M buffer (pH 7.0) at bare GCE (a), at GO/GCE (b), at AuNPs/GO/GCE (c), and at TBABr/SDS⋅⋅⋅AuNPs/GO/GCE (d); scan rate of 50 mV/s. (**B**) Nyquist plots of 0.80 µM DA in 0.1 M buffer (pH 7.0) at bare GCE (a), at GO/GCE (b), at AuNPs/GO/GCE (c), and at TBABr/SDS⋅⋅⋅AuNPs/GO/GCE (d). The frequency range is from 1 Hz to 100 kHz.

The electrochemical behavior of 0.80 µM DA was investigated using DPV with a bare GCE (a), a GO/GCE (b), an AuNPs/GO/GCE (c), and TBABr/SDS⋅⋅⋅AuNPs/GO/GCE (d), as shown in Figure 3A. The DPV of DA showed a broad oxidation peak at 0.42 V on the bare GCE. On the GO/GCE, DA oxidation occurred at 0.40 V, with a negative shift of 0.02 V and an 8.0-fold increase in the peak current compared with the bare GCE, showing the electrocatalytic effect and high surface area of the GO. On the AuNPs/GO/GCE, the DA oxidation potential decreased to 0.37 V, which was a further decrease of 0.03 V compared with the GO/GCE. The extra 1.5-fold increase in the peak current can be associated with the more efficient catalytic effect of AuNPs for DA oxidation. Meanwhile, TBABr/SDS⋅⋅⋅AuNPs/GO/GCE does not cause a significant change in the potential of DA, but leads to significant increase in the anodic peak current of 1.8-fold compared with AuNPs/GO/GCE, because DA could interact with the C–H chain of supramolecular assembly of surfactants (TBABr/SDS) via hydrophobic interaction, thus reaching the surface of the electrode easily. These phenomena demonstrated that the supramolecular electrochemical sensor, TBABr/SDS⋅⋅⋅AuNPs/GO/GCE, could promote the electron transfer of DA at the electrode surface and improve the sensitivity of the electrochemical sensor.

The interfacial properties of the modified electrode surface were studied by electrochemical impedance spectroscopy (EIS). The impedance data was obtained from the modified Randles circuit consisting of the interfacial capacitance (C_dl_) with the parallel combination of the charge transfer resistance (Rct) and the diffusion impedance (*W*) [48]. Figure 3B shows the results for impedance spectrum on a bare GCE (a), a GO/GCE (b), an AuNPs/GO/GCE (c), and TBABr/SDS⋅⋅⋅AuNPs/GO/GCE (d). The electron transfer resistance (R_ct_) at the GCE was estimated to be 254 Ω, which dropped to 45 Ω at the GO/GCE and to 15 Ω at the AuNPs/GO/GCE. Meanwhile, R_ct_ of TBABr/SDS⋅⋅⋅AuNPs/GO/GCE was 10 Ω, providing much lower electron transfer resistance on the surface of TBABr/SDS⋅⋅⋅AuNPs/GO/GCE.

### 2.3. Electrochemical Behavior of DA at Modified Electrodes in the Presence of Supramolecular Assemblies of Mixed Surfactants

To enhance the performance of the electrochemical sensor, the surfactants are an important parameter that can accelerate the diffusion of DA to the surface of the electrode. Several types of mixed surfactants to form the supramolecular assembly were studied including cationic surfactants such as TBABr, DTAB, TTAB, and CTAB, and anionic surfactants such as SDS. It was found that the oxidation peak current of DA was decreased in the presence of supramolecular assemblies of DTAB/SDS, TTAB/SDS, and CTAB/SDS. Meanwhile, the oxidation peak current of DA increased and gave the highest response with the supramolecular assemblies of TBABr/SDS (see Supplementary Data, Figure S1).

To form the supramolecular assemblies of mixed surfactants between TBABr and SDS, the concentrations of surfactants were studied. The concentrations of two surfactants were studied in the range of 0.04–0.12 mM (fixed molar ratio of TBABr/SDS of 1:1), which is lower than its critical micellar concentration (CMC) (CMC of SDS is 8.3 mM and CMC of TBABr is 588 mM) [49,50,51]. The results (Figure S2, see Supplementary) show the oxidation current of DA increased with the increasing TBABr/SDS concentration. At 0.08 mM, TBABr/SDS gave the highest current of DA because SDS is the anionic surfactant, thus the cationic surfactant, which has a small head group, TBABr, could reduce the repulsion of head groups of surfactants [51], and DA could interact with the hydrophobic part of supramolecular assemblies of TBABr/SDS. Above this point, the oxidation current of DA decreased slightly. Thus, the concentrations of both surfactants were chosen at 0.08 mM with the molar ratio of TBABr and SDS of 1:1.

**Figure 4:**
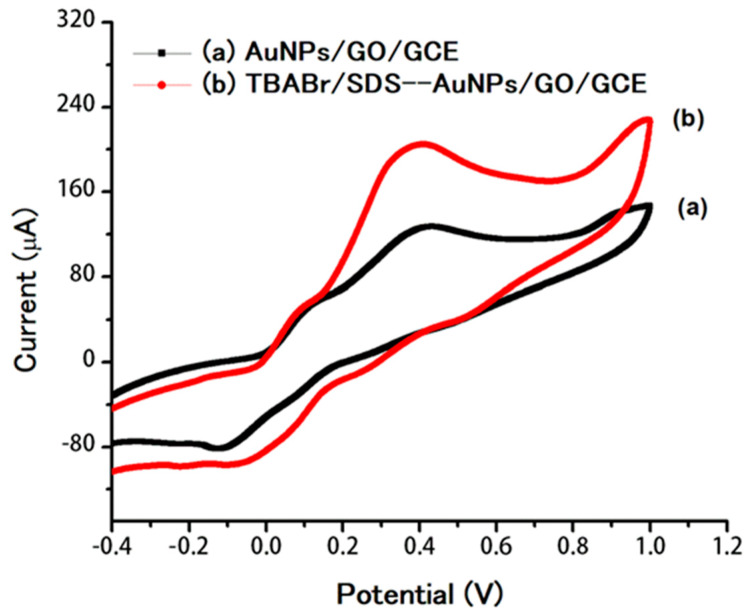
Cyclic voltammograms of 0.80 µM DA in 0.1 M buffer pH 7.0 for AuNPs/GO/GCE at a scan rate of 50 mV/s (**a**) without supramolecular assemblies of TBABr/SDS and (**b**) with 0.08 mM of supramolecular assemblies of TBABr/SDS.

**Figure 5:**
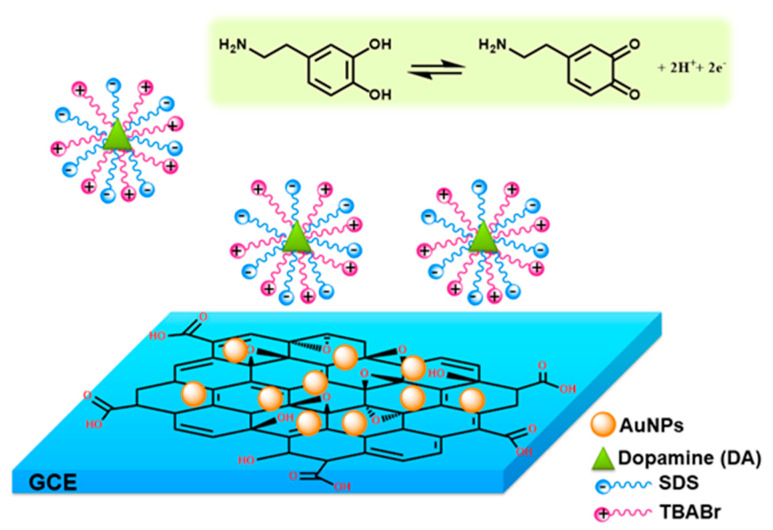
Schematic model illustrating interaction of DA and supramolecular electrochemical sensor as TBABr/SDS⋅⋅⋅AuNPs/GO/GCE.

The comparison of sensitivity of electrochemical sensor (AuNPs/GO/GCE) was tested with and without the supramolecular assemblies of mixed surfactants of the TBABr/SDS system (Figure 4). It was clearly seen that the peak current of DA was increased ca. 1.8-fold in the presence of supramolecular assemblies of the TBABr/SDS system (TBABr/SDS⋅⋅⋅AuNPs/GO/GCE) at 0.08 mM (Figure 4b) when compared with the absence of supramolecular assemblies of the TBABr/SDS system (AuNPs/GO/GCE), as shown in Figure 4a. The surface concentration of the electroactive species (*Γ*) can be calculated by the Laviron equation [52] with the following equation:

$$I{\mathrm{pa}}_{\;}=\frac{n^{2}F^{2}}{4RT}\times \nu A\Gamma$$

where *n* represents the number of electrons involved in the reaction (*n* = 2), A is the surface area of the GCE electrode (0.07 cm^2^), I_pa_ is the peak current of DA (0.8 µM), *Γ* is the surface coverage concentration (mol/cm^2^), *F* is Faraday constant (96,485 C/mol ), and *ν* is the scan rate (50 mV/s). The surface coverage of the electroactive species (*Γ*) can be estimated to be approximately 2.00 × 10^−8^ mol/cm^2^ for the supramolecular system (TBABr/SDS⋅⋅⋅ AuNPs/GO/GCE), which was greater than the *Γ* value with the absence of the supramolecular system (8.36 × 10^−9^ mol/cm^2^). The possible mechanism for the detection of DA using the supramolecular electrochemical sensor (TBABr/SDS⋅⋅⋅ AuNPs/GO/GCE) is described in Figure 5. The interaction of DA and C–H chains of supramolecular assembly of TBABr/SDS via hydrophobic interaction could facilitate the diffusion of electron to the surface of AuNPs/GO/GCE. Thus, the oxidation current of DA was considerably increased in the presence of supramolecular assemblies of mixed surfactants of the TBABr/SDS system.

### 2.4. Effect of pH

**Figure 6:**
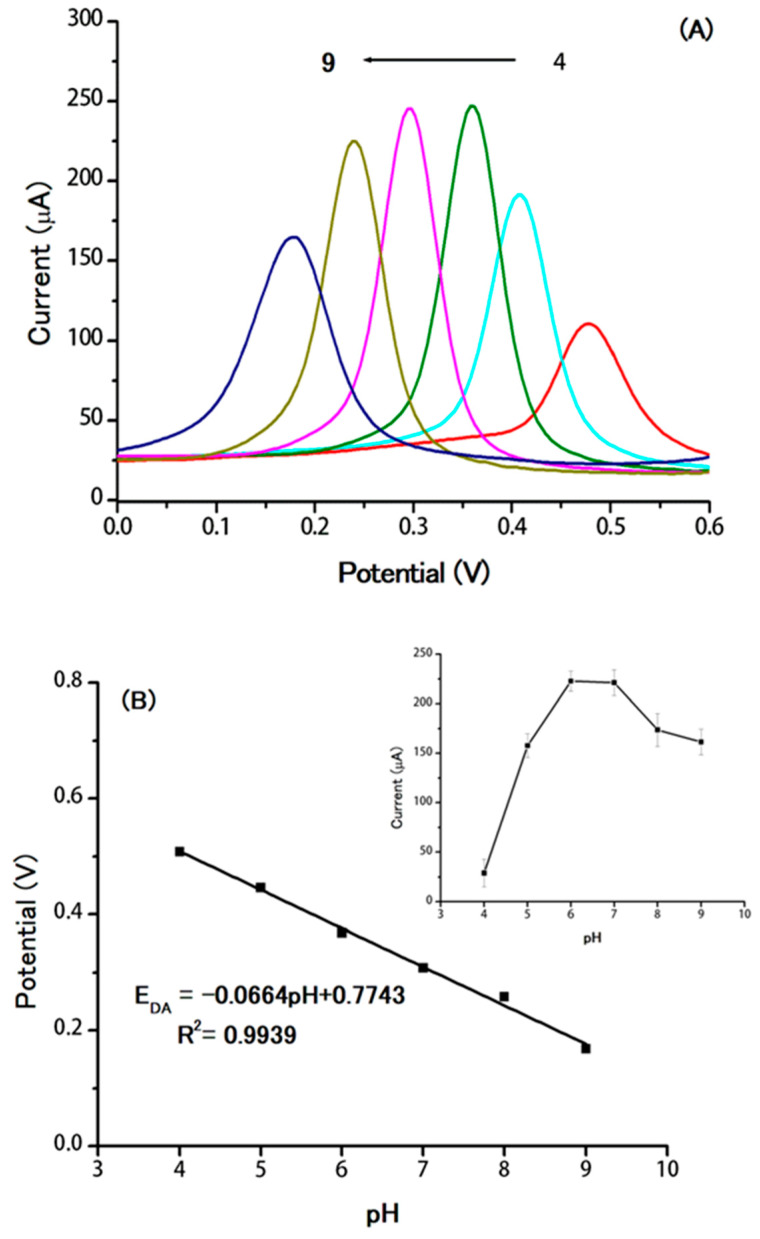
(**A**) Differential pulse voltammograms obtained at the supramolecular electrochemical sensor (TBABr/SDS⋅⋅⋅AuNPs/GO/GCE) in the range of pH 4.0 to 9.0 in 0.1 M PBS containing 0.80 µM DA at a scan rate of 50 mV/s. (**B**) The linear regression equation between potential and pH. Inset: the plot of anodic current of DA versus pH values.

The effect of pH on the oxidation of DA was studied in the range of 4.0–9.0 using the supramolecular electrochemical sensor, TBABr/SDS⋅⋅⋅AuNPs/GO/GCE. As shown in Figure 6, the oxidation peak current of DA increased as the pH solution increased from 4.0 to 7.0 and then decreased at a higher pH solution. Therefore, the optimum pH was selected to be 7.0. With the increase of pH, the oxidation peak potentials of DA are shifted to a more negative potential (Figure 6A), indicating the participation of protons in the electrochemical oxidation of DA [42]. The relationship between pH and potential is described in the following linear equation: E = 0.7743 − 0.0664 pH (R^2^ = 0.9939) (Figure 6B). The obtained slope was 66.4; this values is close to the Nernstian slope (58.6 mV/pH), suggesting an equal number of protons and electrons transfer mechanism [53,54]. To calculate the number of electrons involved in the oxidation of dopamine, the peak width at half height, W_1/2_ = 90/n mV, for the reversible system, where n is the number of electrons in the reaction, was studied [55]. The W_1/2_ was approximately 48 mV over the pH range, suggesting that two electrons are involved in the reaction of dopamine, as proposed in the possible mechanism in Figure 5.

### 2.5. Analytical Performance

**Figure 7:**
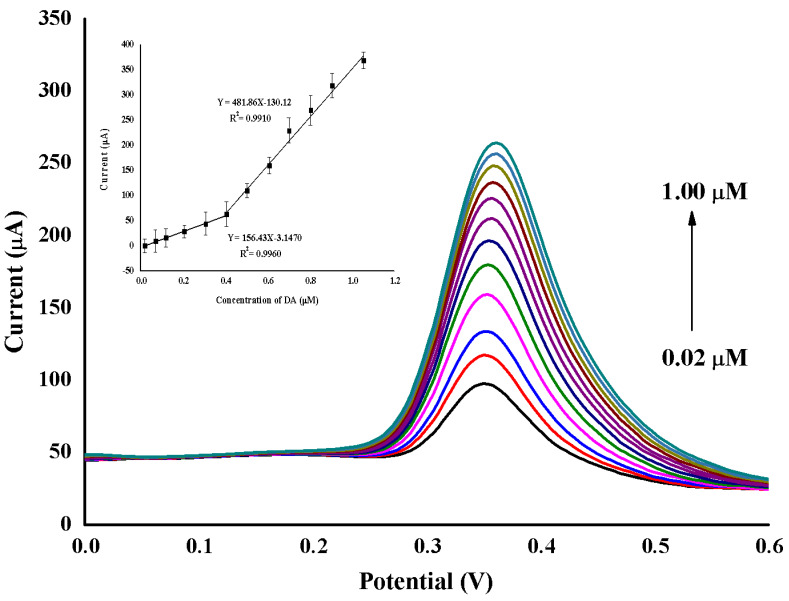
The differential pulse voltammetry (DPV) calibration of DA at the supramolecular electrochemical sensor (TBABr/SDS⋅AuNPs/GO/GCE) in 0.1 M buffer (pH 7.0) at a scan rate of 50 mV/s. Inset: the I_p_ versus concentration.

Method validation was carried out for the determination of DA using the supramolecular electrochemical sensor (TBABr/SDS⋅⋅⋅AuNPs/GO/GCE) at a potential range of 0.0 V to 0.60 V in PBS buffer by DPV. The peak current of DA increased linearly with the increasing DA concentrations in the range of 0.02 µM to 1.00 µM (Y = 156.43 × −3.1470 and Y = 481.86 × −130.12), with a correlation coefficient of 0.9960 and 0.9910, respectively (Figure 7). The detection limit (LOD) and quantification limit (LOQ) were calculated from the following equations: LOD = 3s/b and LOQ = 10s/b, respectively, where *s* is the standard deviation of blank and *b* is the slope of the calibration curve. The LOD and LOQ were found to be 0.01 µM and 0.06 µM, respectively. In addition, the proposed supramolecular electrochemical sensor (TBABr/SDS⋅⋅⋅AuNPs/GO/GCE) is more sensitive and provides a lower LOD than the other published works (Table 1).

The proposed supramolecular electrochemical sensor was applied to detect the DA in standard human serum samples. The standard DA solution was added into diluted human serum (at the ratio of human serum: 0.1 M PBS pH 7.0 of 1:4) with three different concentrations (0.10, 0.20, and 0.30 µM) before the detection. The results are summarized in Table 2, and the recoveries were in the range from 97.26% to 104.21%, which show the TBABr/SDS⋅⋅⋅AuNPs/GO/GCE sensor is reliable for the determination of DA in real samples.

### 2.6. Interferences, Stability, and Reproducibility of the Supramolecular Electrochemical Sensor

To evaluate the selectivity of the supramolecular electrochemical sensor (TBABr/SDS⋅⋅⋅AuNPs/GO/GCE) for the detection of DA, the effect of some commonly existing interferents was studied, including ascorbic acid (AA), uric acid (UA), citric acid, cysteine, lysine, and glucose, which were expected to be found in human serum samples [60]. The interference occurred when current intensity of DA was changed ≥5%. The results indicated that the 500-fold increased concentration of leucine, cysteine, glucose, and sucrose, and 50-fold increased concentration of AA and UA, did not cause significant changes in the current intensity of DA (0.80 µM). It can be concluded that the proposed supramolecular electrochemical sensor was highly selective for the detection of DA.

The peak current of 0.80 µM DA was evaluated for seven measurements (*n* = 7) in real samples using the supramolecular electrochemical sensor as TBABr/SDS⋅⋅⋅AuNPs/GO/GCE. The precision, which is expressed in terms of relative standard deviation (RSD) for the peak current of DA, was found to be less than 3.0% for intra-day and 8.0% for inter-day, indicating the TBABr/SDS⋅⋅⋅AuNPs/GO/GCE electrode had high reproducibility. When the modified electrode was used for 15 days, the peak current of DA at the same concentration was slightly decreased by about 10%, demonstrating that the supramolecular electrochemical sensor showed good stability.

## 3. Experimental Section

### 3.1. Material and Methods

All chemicals used were of analytical-reagent grade and obtained from different sources: dopamine (DA, Sigma-Aldrich, St. Louis, MO, USA), gold (III) chloride trihydrate (HAuCl_4_⋅3H_2_O, Sigma-Aldrich, St. Louis, MO, USA), ascorbic acid (AA, Sigma-Aldrich), uric acid (UA, Sigma-Aldrich), sodium hydroxide (NaOH, Sigma-Aldrich), sodium phosphate monobasic dihydrate (NaH_2_PO_4_·2H_2_O, Sigma-Aldrich), sodium phosphate dibasic (Na_2_HPO_4_, Sigma-Aldrich) and phosphoric acid (H_3_PO_4_, Sigma-Aldrich), potassium hexacyanoferrate (III) (K_3_Fe(CN)_6_, Sigma–Aldrich), citric acid (C_6_H_8_O_7_, Sigma-Aldrich), potassium chloride (KCl, Sigma-Aldrich), N,N-dimethylformamide (DMF, Sigma-Aldrich), l-cysteine (Fluka Chemie AG, Buchs, Switzerland), lysine (Fluka Chemie AG), glucose (Sigma-Aldrich), *N*,*N*-dimethylformamide (DMF, Sigma-Aldrich), sodium dodecyl sulfate (SDS, Fluka Chemie AG), cetyltrimethylammonium bromide (CTAB, Fluka Chemie AG), trimethyltetradecyl ammonium bromide (TTAB, Fluka Chemie AG), dodecyltrimethy–lammonium bromide (DTAB, Sigma-Aldrich), *N*,*N*-dimethylformamide (DMF, Sigma-Aldrich), and tetrabutylammonium bromide (TBABr, Acros Organics). Deionized water (18.2 MΩ) was used in all experiments.

Stock solution of DA (50 mM) was prepared daily by dissolving in water. Phosphate buffers with various pH were prepared by mixing the 0.1 M NaH_2_PO_4_·2H_2_O and 0.1 M Na_2_HPO_4_ at different ratios, and adjusted by adding 1.0 M H_3_PO_4_ solution.

The surface morphology of the modified electrodes was conducted by scanning electron microscopy (Zetasizer Nano S90, Malvern, UK). All electrochemical experiments, including cyclic voltammetry (CV), differential pulse voltammetry, and electrochemical impedance spectroscopy (EIS), were performed on a AutoLab PGSTAT302N (Utrecht, Switzerland) at room temperature. A glassy carbon electrode (GCE) modified with GO and deposited AuNPs (AuNPs/GO/GCE) was used as working electrodes, Ag/AgCl (3 M NaCl) as reference electrode (CorrTest,, Hebei, China), and a platinum wire as counter electrode (CorrTest, Hebei, China) to complete a three-electrode system.

### 3.2. Preparation of the AuNPs/GO/GCE

Graphene oxide was prepared from graphite powder by Hummers’ method with some modifications [61,62]. Five milligrams of GO was dispersed in 5 mL DMF, and then ultrasonicated for 15 min until a homogenous suspension of GO was obtained. GO dispersion (5 µL) was carefully dropped on the top of glassy carbon electrode (GCE), allowing the solvent to evaporate at room temperature (Figure S3, see Supplementary). The GO/GCE was then obtained.

To prepare AuNPs/GO/GCE, the GO/GCE electrode was immersed in 0.1 M H_2_SO_4_ solution containing 1.0 mM HAuCl_4_. The electrochemical deposition of the AuNPs was conducted for 40 s at −1.0 V (Figure S4, see Supplementary). Finally, the modified electrode was cleaned by applying a potential scan of −1.0 V to 1.0 V with a scan rate of 50 mV/s in phosphate buffer solution (pH 7.0) until a steady voltammogram was obtained. Meanwhile, the supramolecular electrochemical sensor, TBABr/SDS⋅⋅⋅AuNPs/GO/GCE, was fabricated using the mixture of TBABr and SDS at 1:1 molar ratio (0.08 mM). The solution was stirred for 5 min before adding 0.20 µM DA.

## 4. Conclusions

A novel and highly sensitive supramolecular electrochemical sensor was developed for the detection of dopamine (DA) based on supramolecular assemblies of mixed surfactants, tetra-butylammonium bromide (TBABr), and sodium dodecyl sulphate (SDS), on gold nanoparticles/graphene oxide modified glassy carbon electrode (TBABr/SDS⋅⋅⋅AuNPs/GO/GCE). The supramolecular assemblies of mixed cationic and anionic surfactants, TBABr and SDS, can increase the electrochemical performance of the determination of DA based on the hydrophobic interaction and electrostatic attraction. The low LOD of DA was displayed as 0.01 µM (3s/b). The proposed supramolecular electrochemical sensor has good sensitivity and stability. In addition, TBABr/SDS⋅⋅⋅AuNPs/GO/GCE was applied to detect DA in human serum samples with satisfactory recovery (97.26–104.21%).

## Figures and Tables

**Table 1:** Comparison of the proposed method with the literature methods for dopamine (DA) determination. TBABr/SDS, tetra-butylammonium bromide/sodium dodecyl sulphate; AuNPs/GO/GCE, gold nanoparticles on graphene oxide modified on glassy carbon electrode; RGO, reduced graphene oxide.

Method	Linear Range (µM)	Correlation Coefficient (R^2^)	Detection Limit (µM)	References
TBABr/SDS⋅⋅⋅AuNPs/GO/GCE	0.02–1.00	0.9960 and 0.9910	0.010	Present work
AuNPs/rGO/Pt wire	0.05–3.0	0.9980	0.016	[56]
AuNPs/Gr/OPPy-MIP/GCE	0.5–8.0	0.9900	0.10	[57]
Cubic Pd/RGO/GCE	0.45–421	0.9968	0.18	[58]
Co(OH)_2_/BAMB/GO	3–20 and 25–100	0.9905 0.9944	0.40	[59]
MWCNTs/EDAS/AuNPs/GCE	0.5–50	0.9968	0.08	[37]
AuNPs/MWCNTs/GCE	0.06–8	0.9984	0.04	[39]
AuNPs/PTAP/GCE	0.15–1.5	0.9925	0.017	[42]

**Table 2:** The recovery of the proposed supramolecular electrochemical sensor for the detection of DA.

Analyte	Human Serum Sample 1 (*n* = 3)			Human Serum Sample 2 (*n* = 3)	
	Spiked (µM)	Found (µM)	Recovery (%)	Found (µM)	Recovery (%)
DA	-	nd *	-	nd *	-
	0.10	0.097	97.26 ± 8.55	0.104	104.21 ± 6.01
	0.20	0.207	103.66 ± 3.41	0.199	99.50 ± 1.33
	0.30	0.303	101.19 ± 3.68	0.302	100.73 ± 2.64

* nd: not detected.

## Supplementary Materials

The following are available online at https://www.mdpi.com/1420-3049/25/11/2528/s1, Figure S1: Effect of types of surfactant for the detection of 0.20 µM DA in 0.1 M buffer (pH 7.0) at AuNPs/GO/GCE, and scan rate of 50 mV/s; Figure S2: Effect of concentration of TBABr/SDS for the detection of 0.20 µM DA in 0.1 M buffer (pH 7.0) at AuNPs/GO/GCE, and scan rate of 50 mV/s; Figure S3: The relationship betweenthe amount of GO and the oxidation peak current of 0.20 µM DA in 0.1M buffer (pH 7.0); Figure S4: The relationship between Au deposition time and the oxidation peak current of 0.20 µM DA in 0.1 M buffer (pH 7.0).
