# Supplementary material for: Supramolecular Electrochemical Sensor for Dopamine Detection Based on Self-Assembled Mixed Surfactants on Gold Nanoparticles Deposited Graphene Oxide
Source: Molecules. 2020 May 29;25(11):2528. doi: 10.3390/molecules25112528 (PMC7321304; doi:10.3390/molecules25112528)
Supplement: Supplementary file 1 [file molecules-25-02528-s001.pdf]

Supporting Information

Supramolecular electrochemical sensor for dopamine detection based on self-assembled mixed surfactants on gold nanoparticles deposited graphene oxide

Pikaned Uppachai[1], Supalax Srijaranai[2], Suta Poosittisak[2], Illyas Md Isa[3], Siriboon Mukdasai[2]*

[1] Department of Applied Physics, Faculty of Engineering, Rajamangala University of Technology Isan, Khon Kaen Campus, Khon Kaen 40000, Thailand

[2] Materials Chemistry Research Center, Department of Chemistry and Center of Excellence for Innovation in Chemistry, Faculty of Science, Khon Kaen University, Khon Kaen, 40002, Thailand

[3] Department of Chemistry, Faculty of Science and Mathemathics, Universiti Pendidikan Sultan Idris, 35900 Tanjong Malim, Perak, Malaysia

* Corresponding author

Tel : +66-43-009700 ext. 42174, 42175

Fax : +66-43-202373

E-mail address: Siriboon Mukdasai (sirimuk@kku.ac.th)

1. Electrochemical behavior of DA at modified electrodes in the presence of supramolecular assemblies of mixed surfactants

To enhance the performance of electrochemical sensor, the surfactants are an important parameter which can accelerate the diffusion of DA to the surface of electrode. Several types of mixed surfactants to form the supramolecular assembly were studied including cationic surfactants such as TBABr, DTAB, TTAB and CTAB, and anionic surfactants such as SDS. It was found that oxidation peak current of DA was decreased in the presence of supramolecular assemblies of DTAB/SDS, TTAB/SDS and CTAB/SDS. While, the oxidation peak current of DA was increased gave the highest response with the supramolecular assemblies of TBABr/SDS (See in supplementary data, **Figure S1**).

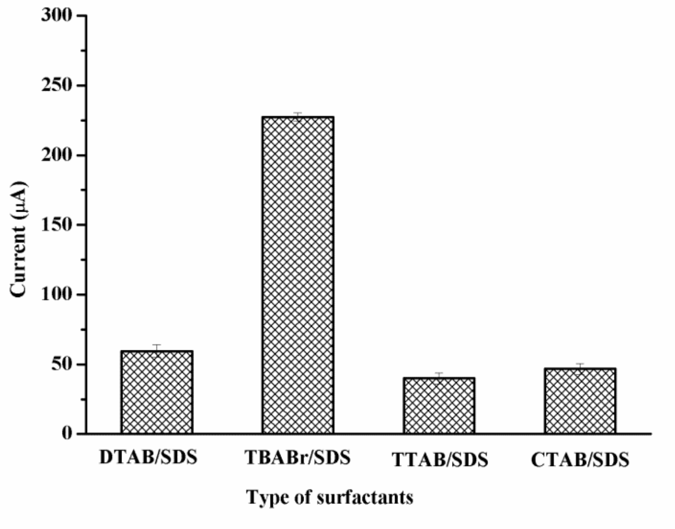

Figure S1 Effect of types of surfactant for the detection of 0.20 μM DA in 0.1 M buffer (pH 7.0) at AuNPs/GO/GCE, and scan rate of 50 mV/s.

To form the supramolecular assemblies of mixed surfactants between TBABr and SDS, the concentrations of surfactants were studied which is lower than its critical micellar concentration or CMC (CMC of SDS is 8.3 mM and CMC of TBABr is 588 mM) [49-51]. Therefore, the concentration of two surfactants were studied in the range of 0.04-0.12 mM (as fixed molar ratio of TBABr:SDS as 1:1). The results (**Figure S2**) showed the oxidation current of DA increased with increasing of TBABr/SDS concentration. At 0.08 mM TBABr/SDS gave the highest current of DA because SDS is anionic surfactant, thus the cationic surfactant which has a small head group, TBABr, could reduce the repulsion of head groups of surfactants [51] and DA could interact with hydrophobic part of supramolecular assemblies of TBABr/SDS. Above this point, the oxidation current of DA was slightly

decreased. Thus, the concentrations of both surfactants were chosen at 0.08 mM with the molar ratio of TBABr and SDS as 1:1.

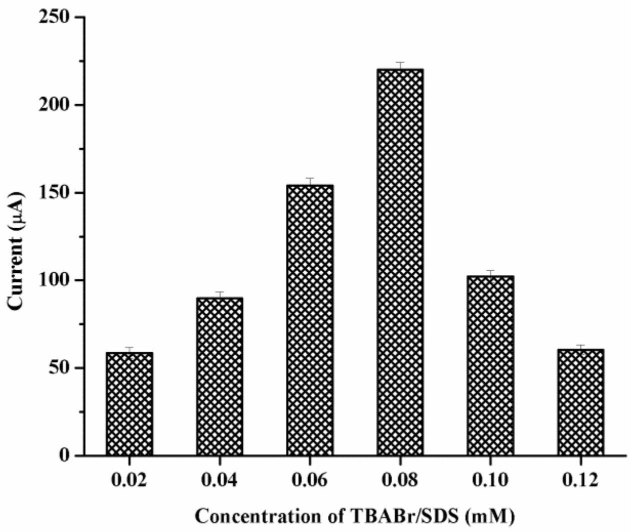

Figure S2 Effect of concentration of TBABr/SDS for the detection of 0.20 μM DA in 0.1 M buffer (pH 7.0) at AuNPs/GO/GCE, and scan rate of 50 mV/s.

2. Effect of the graphene oxide

To optimize the amount of GO on the GCE by DPV, the amount of GO ranging from 1 to 7 μL were studied. **Figure S3** shows that the oxidation peak current of 0.20 μM DA increased with increasing amount of the GO, and reached a maximum at 5 μL, then decreased with the following increase of the GO. This may be due to the thicker film of GO, which prevented the electrical conductivity. Therefore, 5 μL of a GO was used to modify the GCE.

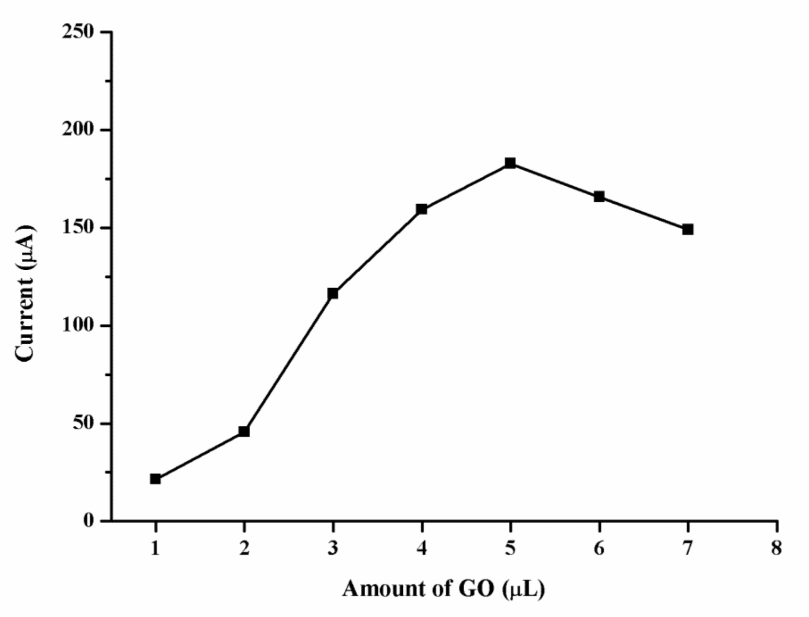

Figure S3 The relationship between the amount of GO and the oxidation peak current of 0.20 μM DA in 0.1M buffer (pH 7.0).

3. Effect of AuNPs deposition time on the GO/GCE

The effect of the deposition time of AuNPs on the peak current of 0.20 μM DA has been studied in the range from 5 s to 50 s and the results are shown in **Figure S4**. The peak current of DA on the AuNPs/GO/GCE increased with AuNPs deposition time at 40 s. However, when the deposition time of AuNPs was longer than 40 s, the peak current dropped slightly, which may due to the aggregation of AuNPs on the electrode surface that can decrease the electrode surface. Therefore, 40 s was selected as the deposition time.

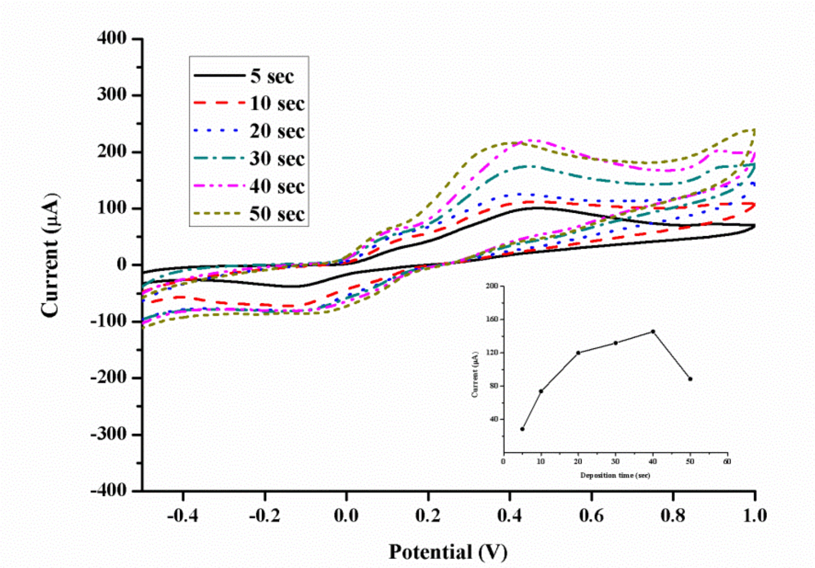

Figure S4 The relationship between Au deposition time and the oxidation peak current of 0.20 μM DA in 0.1 M buffer (pH 7.0).
